# Immune Restoration Syndrome with disseminated *Penicillium marneffei *and Cytomegalovirus co-infections in an AIDS patient

**DOI:** 10.1186/1742-6405-4-21

**Published:** 2007-10-08

**Authors:** Swati Gupta, Purva Mathur, Dipesh Maskey, Naveet Wig, Sarman Singh

**Affiliations:** 1 Division of Clinical Microbiology, Department of Laboratory Medicine, All India Institute of Medical Sciences, New Delhi 110029, India; 2 Department of Internal Medicine, All India Institute of Medical Sciences, New Delhi 110029, India

## Abstract

**Background:**

*Penicillium marneffei *is a dimorphic fungus, endemic in South-east Asia. The fungus causes severe disease in immunocompromised patients such as AIDS. However, no case of immune restoration disease of *Penicillium marneffei *is reported in literature from a non-endemic area.

**Case Presentation:**

We report the first case of *Penicillium marneffei *and Cytomegalovirus infection manifesting as a result of immune restoration one month after initiating HAART. This severely immunocompromised patient had presented with multiple lymphadenopathy, massive hepatosplenomegaly, visual impairment and mild icterus, but no skin lesions. *Penicillium marneffei *was isolated from lymph node fine-needle aspirates and blood cultures.

**Conclusion:**

In order to diagnose such rare cases, the clinicians, histopathologists and microbiologists alike need to maintain a strong index of suspicion for making initial diagnosis as well as for suspecting immune reconstitution syndrome (IRS) with *Penicillium marneffei*.

## Introduction

As a hallmark, all HIV infected patients face severe immune suppression leading to various opportunistic infections. When highly effective antiretrovirals are given to these patients, the main focus of the treating physician is to restore the patient's immune system rapidly. However, while effective immune restoration on one hand achieves immune recovery, it can also be detrimental and lead to worsening of some latent opportunistic infections. This syndrome is known as the immune reconstitution syndrome (IRS) or immune restoration disease (IRD) [1]. The resulting clinical manifestations of this phenomenon are diverse and depend on the associated pathogens viz. mycobacteria, parasites, viruses, or fungi [1,2]. Amongst the fungi, so far, IRS has been extensively reported with *Cryptococcus neoformans *[3], *Histoplasma capsulatum *[4], *Pneumocystis jirovecii *[5] and Aspergillus [6]. To the best of our knowledge, IRS has not yet been reported with *Penicillium marneffei *from a non-endemic region.

*Penicillium marneffei *was first isolated from bamboo rats (*Rhizomys sinensis*) in Vietnam [7]. It is a facultative intracellular pathogen and is capable of causing disseminated infection in both humans and animals. It is endemic in Southeast Asia especially Myanmar, southern China, Thailand, Indonesia, Laos, Malaysia and Vietnam [8]. This organism now represents one of the AIDS-defining pathogens in this region [9] and is reported in up to 20% of HIV infected patients in northern Thailand [10]. *Penicilliosis marneffei *in these patients is usually life threatening, and presents with fever, anaemia, weight loss, and characteristic skin lesions [9,10]. *Penicilliosis marneffei *in Indian HIV patients has also been reported, albeit infrequently, only from Manipur, a north-eastern state of India which shares borders with Myanmar [11].

## Case description

A 35 year old male patient native of Manipur, India, but residing and working in Delhi for the last three years, presented with complaints of fever, loose motions (4–5 times a day), loss of weight and appetite, easy fatigability, pain and heaviness in the abdomen for two months. He had been taking over-the counter drugs with some relief of fever but with reappearance of the present symptoms. A past history of recurrent febrile episodes since two years was also elicited from the patient. On examination, he was found to be alert, thin built, pale, and febrile (temperature 100°F). His systemic examination showed hepatomegaly (two fingers below the right sub costal margin), splenomegaly (four fingers below left sub costal margin) and multiple vesicular lesions over the glans and prepuce. He gave no history of intra-venous drug use. The patient was counselled and after informed consent he was tested positive for HIV-1. His hemogram studies revealed a low haemoglobin (Hb) of 10.0 g/dL with a total leukocyte count TLC of 4400 cells/μL (absolute lymphocyte count: 968/μL; absolute neutrophil count: 2816/μL), a platelet count of 1, 30,000/μL and smear negative for the malarial parasite. His liver function tests were within normal limits. Stool examination on three occasions did not show any pathogenic micro-organism. Three consecutive bacterial blood-cultures were sterile on day seven of incubation at 37°C. Serological tests for malaria, typhoid and kala-azar (rKE-16 antibodies) were also negative. Sputum for acid-fast bacilli was negative on three occasions. Blood was also sent for mycobacterial cultures. Contrast Enhanced Computer Tomography (CECT) chest revealed small round opacity in the posterior-basal segment in the left lung. Ultrasound abdomen showed hepatosplenomegaly without any free fluid. Bone-marrow examination revealed a hypoplastic marrow with lymphoplasmacytosis; suggestive of reactive changes. His CD_4_+ and CD_8_+ T-cell counts were 4 cells/μL and 238 cells/μL, respectively. Based on the NACO guidelines, keeping in view his extremely low CD4+ T-cell count, he was started on highly active antiretroviral therapy (HAART) with three drugs (lamivudine, nevirapine and stavudine) with close follow-up along with prophylaxis for PCP (*Pneumocystis*) and MAC (Mycobacterium-avium complex). He was also started on empirical anti-tubercular therapy (ATT) and given acyclovir for herpes.

At first follow-up after 1 week of therapy, he seemed to tolerate the regimen well. One month later, he came back with complaints of persistent pain abdomen which was associated with progressively increasing loss of appetite, swelling in the axilla and dragging sensation in the abdomen. He was found to be afebrile, but had pallor, mild icterus, cervical (1 cm × 1 cm) and axillary (2 cm × 3 cm, mobile) lymphadenopathy, hepatomegaly and massive splenomegaly extending up to the suprapubic region. He was admitted for detailed investigations. Laboratory investigations at this occasion revealed pancytopenia (Hb 10.1 g/dL; TLC 2200 cells/μL with a differential of 46% polymorphs; 52% lymphocytes, 1% each of eosinophils and monocytes; Platelet count 61,000/μL) though bleeding and clotting times were within normal limits. Blood cultures were sterile after seven days of incubation at 37°C and were discarded thereafter. At this time his liver enzymes were raised (alanine aminotransferase (ALT), 58 IU/L; asparatate aminotransferase (AST), 79 IU/L; alkaline phosphatase (SAP), 669 IU/L) and screening of blood for viral markers on ELISA showed positive HBsAg and HBeAg (*bio Merieux, France*) but HBcIgM and anti-HCV antibodies (DETECT-HCV™) were negative. Viral quantification revealed more than 2,00,000 copies/ml of HBV DNA. His liver enzymes later progressed to ALT, 131 IU/L; AST, 248 IU/L; SAP 2247 IU/L suggesting an infiltrative disease. Blood samples for mycobacterial culture (MGIT 960) came negative by this time and PCR for *Mycobacterium *was also negative. CECT chest revealed bilateral reticulonodular patches in the mid and lower lung zones with mediastinal lymphnodes while CECT abdomen showed massive hepatosplenomegaly along with dilated portal vein and collaterals. Endoscopy of the upper gastrointestinal tract revealed only congestive gastropathy. During the workup, the patient also started complaining of dimness of vision in left eye. He was investigated for other opportunistic pathogens too. A nested-PCR done from urine and blood was strongly positive for cytomegalovirus (CMV). A review of the patient's ophthalmic examination revealed bilateral retinitis typical of CMV with immune mediated uveitis in the left eye. A provisional diagnosis of immune restoration disease (IRD) due to CMV was considered.

**Figure 1:**
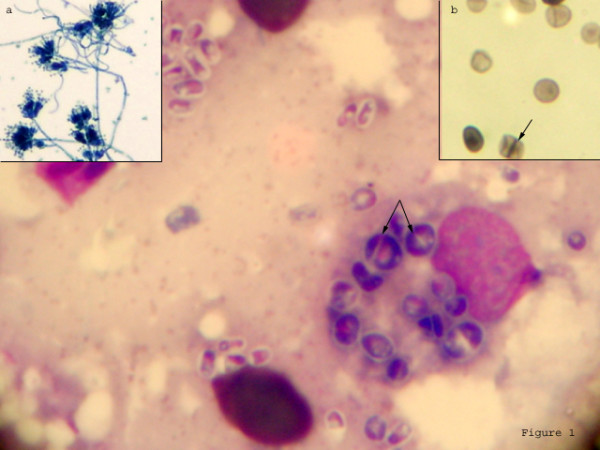
Photomicrograph of Giemsa stained lymph node aspirate showing intracellular as well as extra-cellular yeast cells. Distinctive septation was seen on Gomori's methenamine silver stain (Inset **b**) and also visible as negative staining on Giemsa (arrows). Lacto-phenol cotton blue preparation from growth showed typical *Penicillium *heads (Inset **a**).

To establish a cause of pancytopenia, multiple lymphadenopathy, and hepatosplenomegaly despite the absence of fever, more invasive tests were performed. Bone-marrow biopsy showed a cellular marrow with interstitial infiltrates of plasma cells and multiple granulomas with epitheloid histiocytes. Special stains for fungus and acid fast bacilli were negative. Biopsy of the axillary lymph nodes on haematoxylin-eosin stains revealed follicular lysis with areas of follicular hyperplasia and marked histiocytic proliferation. The histiocytes revealed dot-like fungal elements. However, Giemsa stained smears of the lymph-node aspirate revealed numerous intracellular as well as extra cellular, round, oval and elongated yeast cells (Figure 1). Many of these cells exhibited division by binary fission seen as negative staining on Giemsa, but a prominent septum separating two dividing cells could be ascertained on Gomori's methenamine silver staining (Fig. 1; inset a). No yeast cells were seen with budding. A presumptive diagnosis of *Penicillium marneffei *was made based on the findings of typical septate intracellular and extra cellular yeast cells. A portion of the lymph node aspirate was cultured on a set of duplicate tubes of sabouraud dextrose agar at 25°Celsius and 37°C. Culture at 25°C yielded moist-velvety pink to red colonies with a characteristic intense red pigment diffusing into the medium. Lacto-phenol cotton-blue mounts prepared from the mould-like growths, on light microscopy showed conidiophores bearing chains of conidia characteristic of the *Penicillium *spp. (Fig. 1; inset b). Blood cultures in Brain heart infusion broth was also taken in duplicate sets and showed similar growth after 1 month of prolonged incubation at 25°C.

The final diagnosis of AIDS with chronic hepatitis B, herpes progenitalis and IRS due to *Penicillium marneffei *and Cytomegalovirus was established. His ATT was stopped and he was treated with intravenous amphotericin B (0.6 mg kg^-1 ^day^-1^) for 14 days along with paracetamol/ibuprofen and followed up with oral itraconazole (400 mg day^-1^) for 10 weeks and thereafter on maintenance with 200 mg day^-1^. He was also started on Valgancyclovir (900 mg twice a day) for 21 days for CMV along with intravitreal corticosteroids in left eye and subsequently maintained on 900 mg day^-1^. His ART was modified to Tenofovir, Lamivir and Efavirin due to derangement in liver functions. He responded well to treatment with regression of lymph nodes and decrease in the size of liver and spleen. After 10 months of therapy, he has gained 20 kg weight, his vision is 6/6 in both eyes and his liver and spleen are not palpable, his hemogram shows Hb 13.2 g/dL, TLC 6300/μL, platelet counts 1,69,000/μL. HBV DNA levels has also decreased to 10^3 ^copies/ml. His repeat CD_4_+ and CD_8_+ T-cell counts were 224 and 470 cells/μL respectively.

## Discussion

Immune reconstitution syndrome (IRS) usually occurs in patients on HAART due to effective inflammatory response to residual pathogens. It is reported that within 4–6 weeks of initiation of HAART, the HIV-RNA load declines while CD_4_+ T-cell count starts increasing [2]. This leads to a paradigm shift of immune response from TH 2 type to TH1 type. Patients with very low CD_4_+ T-cell count and high HIV viral load are more prone for IRS particularly with intracellular pathogens. IRS is now a major concern in developing countries where aggressive HAART therapy is now easily available. In this case, the patient began deteriorating clinically with development of unusual symptoms 4 weeks after initiating HAART. His symptomatology included multiple lymphadenitis, massive hepatosplenomegaly and visual defects in the absence of fever. Biopsy of the lymphnodes as well as bone marrow revealed multiple epitheloid granulomas with histiocytic infiltration indicating an active immune response. Opthalmoscopic examination also revealed immune mediated uveitis in the left eye which was not present prior to therapy. The patient showed a good response to antiretroviral therapy with a rise in his CD4^+ ^T-cell count. We were unable to get the HIV viral load of this patient due to financial constraints. Even though no clear cut definition for IRS has been laid down, yet these features are consistent with the proposed criteria for diagnosis of an IRS as reported earlier [1,2,12]. Before starting HAART, this patient did not have any manifestations suggestive of penicilliosis such as lymphadenopathy, massive hepatosplenomegaly and severe pancytopenia. All these point towards an atypical exuberant inflammatory response rather than secondary to the immunodeficient state. IRS with fungal pathogens like *Cryptococcus *and *Histoplasma *also usually present with lymphadenitis [2]. While *Histoplasma *may also present with uveitis [2], *Cryptococcus *usually presents with recurrent meningitis [12]. The hallmark lesion in these cases has been the presence of granulomas with or without fungal elements.

To the best of our knowledge, ours is the first case of IRS with *Penicillium marneffei *outside an endemic area with atypical symptoms presenting for the first time only 4–6 weeks after initiation of HAART. Here, it was a case of unmasking of a previously quiescent or latent infection probably acquired long back when the patient had visited his native village in Manipur, in North-east India. *Penicillium marneffei *now represents one of the most common AIDS-defining opportunistic infections in endemic areas of Southeast Asia. In India, the infection is endemic only in bamboo cultivation areas of Manipur, a state which shares borders with Myanmar. Diagnosis is aided by the presence of characteristic skin lesions which may be seen in around 81% of patients with Penicilliosis. Though such lesions are not diagnostic for penicilliosis, they are an important clue which aids in rapid diagnosis. The patient in our report was also found to have originated from Manipur but he did not have any skin lesion. Infections with *Penicillium marneffei *in HIV patients have been reported from endemic areas usually late in the course of HIV infection, with a CD_4_+ T-cell count below 50 cells/μL [8]. But, when such an infection presents atypically as an IRS in a non-endemic area, it can be very challenging for diagnosis. This case emphasizes on the varied and uncommon clinical presentations that ought to be understood by the AIDS treating as well as the laboratory physicians. Lastly, the final diagnosis of disseminated penicilliosis could be clinched only after FNA-cytology and prolonged culture of the blood samples and the lymph node aspirate. Therefore, in order to diagnose such conditions, the clinicians, histopathologists and microbiologists alike need to maintain a strong index of suspicion for making initial diagnosis as well as for suspecting IRS with rare fungal pathogens.

## Competing interests

The author(s) declare that they have no competing interests.

## References

[B1] Murdoch DM, Venter WDF, Van Rie A, Feldman C (2007). Immune reconstitution inflammatory syndrome (IRIS): review of common infectious manifestations and treatment options. AIDS Res Ther.

[B2] Singh N, Perfect JR (2007). Immune reconstitution syndrome associated with opportunistic mycoses. Lancet Infect Dis.

[B3] Shelburne SA, Darcourt J, White AC, Greenberg SB, Hamill RJ, Atmar RL, Visnegarwala F (2005). The role of immune reconstitution inflammatory syndrome in AIDS-related Cryptococcus neoformans disease in the era of highly active antiretroviral therapy. Clin Infect Dis.

[B4] Breton G, Adle-Biassette H, Therby A, Ramanoelina J, Choudat L, Bissuel F, Huerre M, Dromer F, Dupont B, Lortholary O (2006). Immune reconstitution inflammatory syndrome in HIV-infected patients with disseminated histoplasmosis. AIDS.

[B5] Koval CE, Gigliotti F, Nevins D, Demeter LM (2002). Immune reconstitution syndrome after successful treatment of *Pneumocystis carinii *pneumonia in a man with human immunodeficiency virus type 1 infection. Clin Infect Dis.

[B6] Sambatakou H, Denning DW (2005). Invasive pulmonary aspergillosis transformed into fatal mucous impaction by immune reconstitution in an AIDS patient. Eur J Clin Microbiol Infect Dis.

[B7] Nelson KE, Kaufman L, Cooper CR, Merz WG (1999). *Penicillium marneffei*: An AIDS-related illness from Southeast Asia. Infect Med.

[B8] Sirisanthana T, Supparatpinyo K (1998). Epidemiology and management of penicilliosis in human immunodeficiency virus-infected patients. Int J Infect Dis.

[B9] WHO-SEARO Publications on HIV/AIDS 2006 (2006). WHO case definitions of HIV for surveillance and revised clinical staging and immunological classification of HIV-related disease in adults aged 15 years or older. WHO-SEARO.

[B10] Supparatpinyo K, Khamwan C, Baosoung V, Nelson KE, Sirisanthana T (1994). Disseminated *Penicillium marneffei *infection in Southeast Asia. Lancet.

[B11] Ranjana KH, Priyokumar K, Singh TJ, Gupta C, Sharmila L, Singh PN, Chakraborti A (2002). Disseminated *Penicillium Marneffei *infection among HIV-infected patients in Manipur state, India. J Infect.

[B12] French MA, Price P, Stone SF (2004). Immune restoration disease after antiretroviral therapy. AIDS.

